# CD33 as a leukocyte-associated marker expressed on human spermatozoa

**DOI:** 10.1186/s13104-023-06324-z

**Published:** 2023-04-20

**Authors:** Nasrin Sereshki, Mitra Rafiee, Razieh Alipour, Kourosh Rahimyan, David Wilkinson

**Affiliations:** 1 Department of Immunology, School of Medicine, Isfahan University of Medical Sciences, Isfahan, Iran; 2 Department of Immunology, Cellular and Molecular Research Center, Birjand University of Medical Sciences, Birjand, 9717853577 Iran; 3 Department of Microbiology, Faculty of Medicine, Shahid Beheshti University of Medical Sciences, Tehran, Iran; 4 University of Aberdeen, Scotland, UK

**Keywords:** Spermatozoa, CD33, Siglecs

## Abstract

**Objective:**

Sialic acid-binding immunoglobulin-type lectins (Siglecs) are commonly present on immune cells and often mediate cell-to-cell interactions and signaling. Studies have shown the presence of Siglecs 1, 2, 5, 6, 10 and 14 on human spermatozoa. To the best of our knowledge, the expression of CD33 on spermatozoa has not yet been studied. Semen samples were collected from 25 healthy men with normal semen status. CD33 expression on purified spermatozoa was evaluated by flow cytometry methods.

**Results:**

The results demonstrate the expression of CD33 on the surface of purified spermatozoa. The mean (± SD) of MFI (mean fluorescence intensity) was 12.85 (± 1.33) and the mean percentage of spermatozoa that express CD33 was 73.75 (± 3.75). Results were obtained showing that spermatozoa express CD33 (or Siglec-3) on their surface. The physiological role of these molecules on spermatozoa remains to be determined. It is recommended that further research be undertaken regarding the role of Siglecs (such as CD33) on spermatozoa apoptosis.

## Introduction

Sialic acid is an essential component of the spermatozoa glycocalyx and is involved in functions of spermatozoa including motility, migration and interaction with cumulus-oocyte complex (COC) [1]. Sialic acids that bind to the end of glycans on the surface of cells and secrete glycoconjugates to form sialoglycans are ligands for sialic acid-binding immunoglobulin-like lectins (Siglecs) [2, 3].

Siglecs are immunoglobulin-type transmembrane proteins and comprise; (a) V-set domain (sialic acid-binding N-terminal), (b) C2-set domain (variable numbers of Ig domains), (c) a transmembrane region and (d) a cytosolic tail [1]. Siglecs are commonly present on immune cells and often mediate cell-to-cell interactions and signaling [4–6]. They transmit inhibitory or activating signals based on possessing ITIM or ITAM on the cytosolic tail [4–6]. Siglecs bind to their ligands expressed on other cells (in trans) in order to communicate with neighboring cells and also interact with ligands expressed on the same cell (in cis) [7].

There are two primary subsets of Siglecs based on their sequence similarities and evolutionary conservation; (a) conserved Siglecs, including Siglecs 1, 2, 4 and 15 and (b) rapidly evolved Siglecs, including Siglec-3 (CD33) in humans and Siglecs 5, 6, 7, 8, 9, 10, 11, 14 and 16 [3]. One of the main roles of some of these receptors is the fertilization process [1, 8, 9]. Studies have shown the presence of Siglecs 1, 2, 5, 6, 10 and 14 on human spermatozoa [1].

To the best of our knowledge, the expression of CD33 on spermatozoa has not yet been studied. CD33 preferentially binds to α2-6- and α2-3-sialylated glycans on the surface of normal and leukemic cells [7, 10]. CD33 is mostly expressed on myeloid cells and on some lymphoid cells, such as NK cells [7]. Studies have shown that CD33 is an inhibitory receptor and therefore has a role in immune regulation [11]. We discovered that CD33 expresses on spermatozoa following inadvertently pouring anti-CD33 antibody (instead of the intended antibody) during an experiment on a semen sample. Therefore, this study seeks to address CD33 expression on the surface of human spermatozoa. The results of this study can be beneficial regarding the use of spermatozoa as a model to study the biological function of CD33 molecule.

## Methods and materials

### Subjects

Twenty-five healthy volunteers aged 25–56 years entered the study. Semen samples were collected by masturbation after 2–3 days of sexual abstinence. After semen analysis according to WHO standard guidelines (WHO, 2010), samples with normal quality (according to WHO reference intervals for values of semen parameters) were selected for the assessment of CD33 expression. Informed consent was obtained from all subjects who participated in this study. The protocol for this study was approved by the Ethics Committee of Isfahan University of Medical Sciences (Isfahan, Iran). The ethics committee approval letter number is IR.MUI.REC.1395.3.480.

### Purification of spermatozoa

Density-gradient centrifugation technique was used for purification of spermatozoa. The procedure of purification is described in more detail elsewhere [12]. In brief, 1ml of the spermatozoa suspension was carefully layered over a discontinuous gradient made by AllGrad 95% and 45%. After centrifugation at 400 g for 18 min, the spermatozoa pellet at the bottom of the centrifuge tubes was washed and re-suspended in AllGrad Wash. The purified spermatozoa were assessed by microscopic visualization for lack of non-spermatozoa cell contamination and viability.

### Flow cytometry

The presence of CD33 on the surface of spermatozoa was measured by direct immunofluorescence using a BD FACS Calibur (BD Biosciences, USA) flow cytometer. 1 × 10^6^ spermatozoa were stained with phycoerythrin (PE) mouse anti-human CD33 (clone: MCD5, IQ Products, Groningen, Netherlands) at room temperature for 30 min and then run on flow cytometry. Data from 100,000 events were collected using Cell Quest software (Becton Dickinson). Antibody titration was performed and the optimal titer with the minimal background was selected. Unstained control was used as a negative control. Cell viability tests were not performed because abnormal and dead spermatozoa were removed by AllGrad solution before staining. FlowJo software version X was used for the data analysis.

## Results

**Fig. 1:**
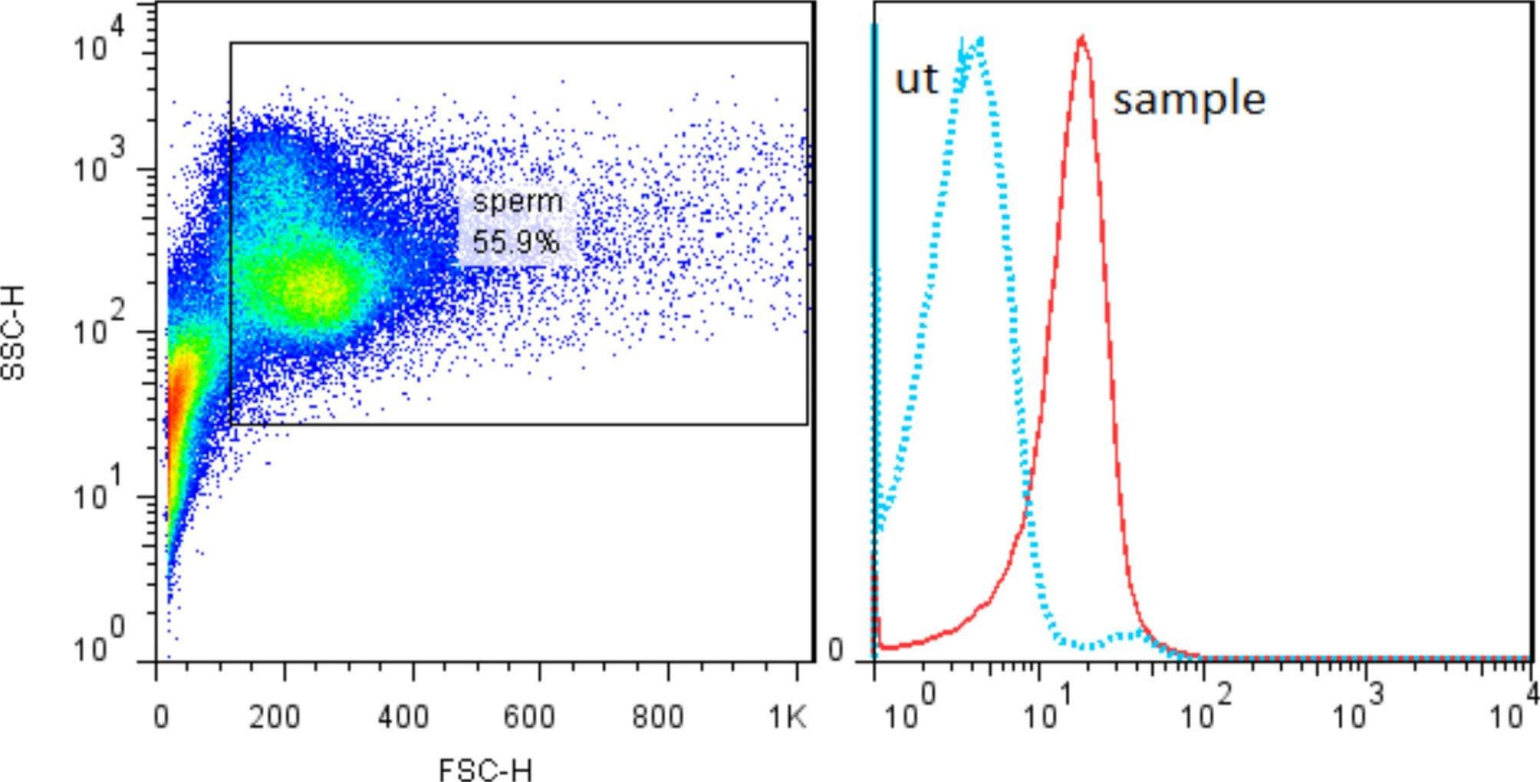
Representative flow cytometry gating plots and histogram of unstained (ut) control and sample. Unstained control was used to differentiate the negative and positive population

CD33 expression on spermatozoa was evaluated by flow cytometric assay. A logarithmic mode of the side scatter (SSC) parameter versus a liner mode of the forward scatter (FSC) parameter was used to detect spermatozoa and a fitting gate was set around them. The corresponding histogram was used to determine the expression of CD33. The results demonstrate the expression of CD33 on the surface of purified spermatozoa (Fig. 1). The mean (± SD) of MFI (mean fluorescence intensity) was 12.85 (± 1.33) and the mean percentage of spermatozoa that express CD33 was 73.75 (± 3.75).

## Discussion

CD33 (Siglec-3) is an inhibitory sialoadhesin receptor that is expressed by human leukocytes [6, 11] and has major roles in the regulation of these cell functions. Interestingly, this study revealed that CD33 (as a leukocyte marker) is expressed on the surface of human spermatozoa. We could not find any study in agreement with, or contrary to, our result and, to the best of our knowledge, this is the first time that the expression of CD33 on spermatozoa has been demonstrated.

The role of other Siglecs expressed on spermatozoa has been studied. It has been determined that the interaction of Siglecs and sialic acids on spermatozoa and the cumulus-oocyte complex (COC) plays the main role in regulating the process of fertilization and embryo development [1, 9, 13].

An in vitro study showed that removing sialic acid from spermatozoa resulted in decreased motility and mucus penetration, but increased zona pellucida binding and polyspermy [1]. Given the currently known functions and structure of Siglects, one can also suppose that Siglecs, including CD33, on spermatozoa may bind to cis ligands (bind to sialic acids on spermatozoa) and lead to the transmission of signals that govern and regulate spermatozoa functions such as motility, penetration, capacitation and acrosome reaction. Obviously, further well-designed studies should be done to confirm or reject this hypothesis.

Studies have shown that CD33 related Siglecs (rapidly evolved Siglecs mentioned in the Introduction) can induce apoptosis [2]. Apoptosis is a process in which the contents of the cell are wrapped into a small package of membrane that these apoptotic bodies are removed by immune cells without induction of inflammation [14]. Previous studies have shown that the crosslinking of CD33 by monoclonal antibody induces apoptosis in AML cells and inhibits the in vitro proliferation of both normal myeloid cells and chronic myeloid leukemia [15]. Additionally, we know from the litreature that spermatozoa apoptosis is essential to remove DNA-damaged cells and prevent the fusion of these damaged cells with oocyte [16]. Thus, the investigation of the probable role of Siglecs (such as CD33) in spermatozoa apoptosis could be an intriguing subject for future researches in this field. It is recommended that further research should be undertaken regarding the role of Siglecs (such as CD33) on spermatozoa apoptosis.

## Conclusion

We present data from antibody labeled FACS showing that spermatozoa express CD33 (or Siglec-3) on their surface. The physiological role of this molecule on spermatozoa remains to be determined. It is recommended that further research should be undertaken regarding the role of Siglecs (such as CD33) on spermatozoa apoptosis.

### Limitations

There were some limitations in this study that are suggested for improving future studies. These limitations include 1 In our study we didn’t use Real-time PCR so we can’t understand whether spermatozoa absorbed CD33 from the environment or it is expressed by itself. 2 Limitation in sample size 3 No comparison of normal samples with abnormal samples.

## Data Availability

The datasets used and/or analysed during the current study are available from the corresponding author on reasonable request.
